# The reasons hypoactive delirium is often overlooked: a reply

**DOI:** 10.1111/anae.70035

**Published:** 2025-10-10

**Authors:** Ben Gibbison, Maria Pufulete

**Affiliations:** ^1^ University of Bristol Bristol UK; ^2^ University Hospitals Bristol and Weston NHS Trust Bristol UK

We thank Li et al. [1] for their interest in our survey [2]. We agree that hypoactive delirium in the ICU is difficult to diagnose, often missed and leads to poor outcomes for patients. It would not be appropriate to cross‐tabulate results as Li et al. suggest. The survey was designed to capture ICU‐level practice and was completed by one individual on behalf of the whole ICU. The training questions reflect ICU‐level practice across different healthcare professions, while the confidence questions reflect belief at the individual level from a person already experienced in delirium care.

The survey was part of a package of projects funded by the UK National Institute of Health and Care Research. In addition to the survey, we conducted a qualitative study [3], a psychometric review of all instruments to detect delirium and systematic reviews of interventions and implementation methods to prevent and manage it [4, 5]. We are currently triangulating these different strands of evidence, which will then contribute to addressing some of the questions posed by Li et al. Our triangulation of the evidence does not support training in the CAM‐ICU as the most important means of improving the detection and treatment of hypoactive delirium. The survey and qualitative study show that the CAM‐ICU is implemented variably, and training is only a small part of why this occurs. There are systemic issues which need to be addressed, and these include enhanced training and awareness; protocol‐driven care; audit and feedback; computer‐generated prompts in clinical information systems; and the inclusion of families in the diagnostic and care processes.

The psychometric review answered the question of whether the CAM‐ICU (or indeed any other tool or instrument) possessed the necessary characteristics to detect or diagnose ICU delirium. This would answer a question earlier in any logic model, that is, are we using the right tool for the job – not just can we train people in it. These data have not yet been published, but we do not believe that increasing training in the CAM‐ICU would improve the detection of any ICU delirium or hypoactive delirium specifically.

Little has changed in over 20 years regarding the evidence for interventions to prevent, detect and manage ICU delirium or in the implementation of, or the adherence to, these tools and interventions [3, 6]. A rethink is required to improve patient care and, therefore, clinical outcomes that matter to patients and healthcare providers. Addressing the systemic issues, approached with behavioural science to make it easy for healthcare staff to provide the ‘right’ care that prevents, diagnoses and manages delirium would be the best way to improve care on ICUs.

## References

[1] Li S , You L . The reasons hypoactive delirium is often overlooked. Anaesthesia 2026; 81: 445–446. 10.1111/anae.16785. 40947830

[2] Gibbison B , Johnson AF , Rowan KM , et al. Delirium identification, prevention and management in intensive care units in England, Wales and Northern Ireland: a survey of practice. Anaesthesia 2026; 81: 41–50. 10.1111/anae.16728. 40790899 PMC12747587

[3] Parslow RM , Gibbison B , Pufulete M , et al. Barriers and facilitators to the delivery of delirium care in intensive care units: an analysis informed by the Theoretical Domains Framework. Anaesthesia 2026; 81: 213–221. 10.1111/anae.70017. 41055047 PMC12803597

[4] Jones KL , Kundakci B , Booth A , Pufulete M , Gibbison B . Protocol for a meta‐review of interventions to prevent and manage ICU delirium. BMJ Open 2025; 15: e090815. 10.1136/bmjopen-2024-090815. PMC11815468 39933812

[5] Kundakci B , Jones KL , Booth A , Parslow RM , Moore AJ , Gibbison B , Pufulete M . Factors contributing to the implementation of interventions to prevent and manage intensive care unit delirium: a systematic review protocol. BMJ Open 2025; 15: e093338. 10.1136/bmjopen-2024-093338. PMC12039013 40295133

[6] Ishii K , Kuroda K , Tokura C , et al. Current status of delirium assessment tools in the intensive care unit: a prospective multicenter observational survey. Sci Rep 2022; 12: 2185. 10.1038/s41598-022-06106-w. 35140285 PMC8828828

